# FastProNGS: fast preprocessing of next-generation sequencing reads

**DOI:** 10.1186/s12859-019-2936-9

**Published:** 2019-06-17

**Authors:** Xiaoshuang Liu, Zhenhe Yan, Chao Wu, Yang Yang, Xiaomin Li, Guangxin Zhang

**Affiliations:** 1 Megagenomics Corporation, Beijing, China; 2 National Renewable Energy Laboratory CO, Colorado, USA; 3 Ping An Health Technology, Beijing, China; 4 NLP, R&D Suning, Beijing, China

**Keywords:** Quality control, Adapter removing, NGS

## Abstract

**Background:**

Next-generation sequencing technology is developing rapidly and the vast amount of data that is generated needs to be preprocessed for downstream analyses. However, until now, software that can efficiently make all the quality assessments and filtration of raw data is still lacking.

**Results:**

We developed FastProNGS to integrate the quality control process with automatic adapter removal. Parallel processing was implemented to speed up the process by allocating multiple threads. Compared with similar up-to-date preprocessing tools, FastProNGS is by far the fastest. Read information before and after filtration can be output in plain-text, JSON, or HTML formats with user-friendly visualization.

**Conclusions:**

FastProNGS is a rapid, standardized, and user-friendly tool for preprocessing next-generation sequencing data within minutes. It is an all-in-one software that is convenient for bulk data analysis. It is also very flexible and can implement different functions using different user-set parameter combinations.

**Electronic supplementary material:**

The online version of this article (10.1186/s12859-019-2936-9) contains supplementary material, which is available to authorized users.

## Background

With the development of next-generation sequencing (NGS) technologies, the cost of sequencing has decreased a lot. From data from the NHGRI Genome Sequencing Program (GSP), over the past seventeen years, the cost of DNA sequence per megabase has decreased from nearly $10,000 to less than $0.1 and the cost of sequencing per human-sized genome has decreased from 100 million dollars to almost one thousand dollars, thus stimulating the production of an avalanche of data. Among the sequencing platforms, Illumina platforms dominate the sequencing market (personal communication, Timmerman, L.). However, because of the nature of the its technology, the average error rate of Illumina Miseq system is nearly 1% [1], which is significantly higher than the error rate of traditional Sanger sequencing platforms, which is below 0.001% [2]. To achieve reliable conclusions from downstream analyses such as variant discovery and clinical applications, quality control (QC) processes like filtration of low quality reads are important. In addition, adapter trimming is essential in most cases because adapter contamination can lead to NGS alignment errors and an increased number of unaligned reads, especially for small RNA sequencing.

To date, several tools with different functions have been developed to deal with FASTQ files produced by Illumina sequencing (Table 1). Some offer only a few functions to deal with FASTQ or FASTA files, such as Seqtk [3], FastQC [4] and PIQA [5]. They are suitable for quality checks but not for preprocessing of NGS reads. PRINSEQ [6], FASTX-Toolkit [7] and NGS QC Toolkit [8] behave poorly on run-time performance. Fastp [9] and a new version of FaQCs [10] are written in C++, but they can’t totally satisfy the demands of different users. We will discuss about it in the following section. Therefore, there is still a great need for a highly efficient user-friendly tool that can preprocess NGS sequencing data in most cases. We have developed FastProNGS, a one-button and all-in-one tool for QC and adapter removal that can handle both single-end and paired-end sequences. Importantly, the efficiency of FastProNGS is much higher than almost all the current methods. We believe FastProNGS will greatly facilitate the preprocessing tasks with its fast and versatile functionality.

**Table 1:** Tools developed for processing next-generation sequencing data

Software	Major functions	Programming language
FastQC	Quality check	Java
PIQA	Quality check	R,C++
FASTX-Toolkit	A collection of tools to filter low quality reads and remove adapters	C,C++
Fqtools	FASTQ file manipulation, such as validate FASTQ and trim reads in a FASTQ file	C
seqtk	Toolkit for processing sequences in FASTA/Q formats, such as format conversion and subsampling of sequences	C
PRINSEQ	Filter, reformat and trim sequences	Perl, R
Multiqc	Aggregate results across many samples into a single report	Python
NGS QC Toolkit	Filter low quality reads and remove adapters.	Perl
Fastp	Filter low quality reads and trim adapters	C++
FaQCs	Filter low quality reads and trim adapters	C++

## Implementation

FastProNGS is written mainly in C and was developed on a Redhat Linux system. To create graphical charts in the report in HTML format, a JavaScript plug-in is included. FastProNGS implements a producer–consumer pattern to support multithreading. In the multithreaded mode, a single file is divided into parts and processed in parallel using multiple CPUs. FASTQ format is used for sequence data input and output and both can be gzip-compressed, which significantly reduces disk storage requirements and is compatible with several downstream analysis tools. FastProNGS supports single-end or paired-end sequences, as well as 33-based or 64-based quality score encodings. To better support downstream analyses, the output can also be obtained as several split files. Furthermore, considering different data sources and requirements for QC, all the parameters in FastProNGS can be used separately. For example, it can be used only to filter low quality reads without removing adapters, depending on the users’ requirements.

### Removing adapters

The alignment algorithm for adapter trimming is an extension of the semiglobal alignment algorithm with the cost function modified to achieve the desired effect of allowing arbitrary overlaps, which is similar to Cutadapt. Parameter –O or --overlap is the minimum overlap parameter between a read and an adapter which can be set to a value higher than the default of three to avoid discarding too many reads with random adapter matches. FastProNGS first searches for an exact match of the adapter sequence within the read sequences. If a match is found, the alignment procedure can be stopped. Auxiliary matrices in the dynamic programming algorithm are used to calculate the alignment error rate, which is defined as the number of errors divided by the number of aligned adapter bases. Only alignments below a given error rate threshold are found, which is defined by parameter –e or --error-rate. The calculation would be stopped at a column when the number of encountered errors exceed the threshold. FastProNGS can also map with indels and approve mismatches between a read and an adapter by default. If parameter –i or --no-indels is defined, only mismatches in alignments are allowed. Multiple adapter removal and 5′ or 3′ adapter trimming in the anchored or non-anchored case are also supported by using parameters such as –b and –B. Once the adapters are found within the sequences, they will be removed directly.

### Quality control and filtering

FastProNGS can perform read quality assessment and filtration based on GC content, occurrence of Ns, read length, and quality scores. Generally, the quality of bases at the ends of reads is substantially lower than the quality of the other bases, so it is crucial to trim these bases to improve the overall quality score of the reads. FastProNGS allows users to trim sequences containing low quality scores (parameter –Q or --quality-low) or Ns (parameter –n or --trim-n) at their 5′- and 3′-ends. Besides, if the total quality score of a read is under a user-defined threshold value (parameter –q or --quality-filter) or the number of Ns exceeds a defined value (parameter –r or --ratio-n), the read will be discarded. Reads that are not within a specified length range after trimming can also be removed, which is defined by parameters --min-length and --max-length. During the filtering process, it is crucial to maintain the pairing information, which is important for downstream analyses. In the process of trimming and adapter removal, paired sequences are processed sequentially. But in the filtering process, each paired reads are analyzed simultaneously. Thus, for paired-end reads, if only one end passes the filtration, both ends will be removed. Trimming is performed before adapter removal and filtering is done after adapter removal.

### Summary and statistics

The quality statistics of the input and output filtered data can be obtained in plain-text, JSON, or HTML format files for different downstream analyses. The statistics include the percentages of bases A, T, C, G, and N; adapter information on the reads; sequence length distribution; and number and percentage of reads filtered at each step. Graphs showing this information are exhibited with a JavaScript plug-in in the HTML report. The plots are editable by Chart Studio and are also resizable, interactive, and can be downloaded in a range of publication-ready formats.

## Results

### Workflow demonstration

**Fig. 1:**
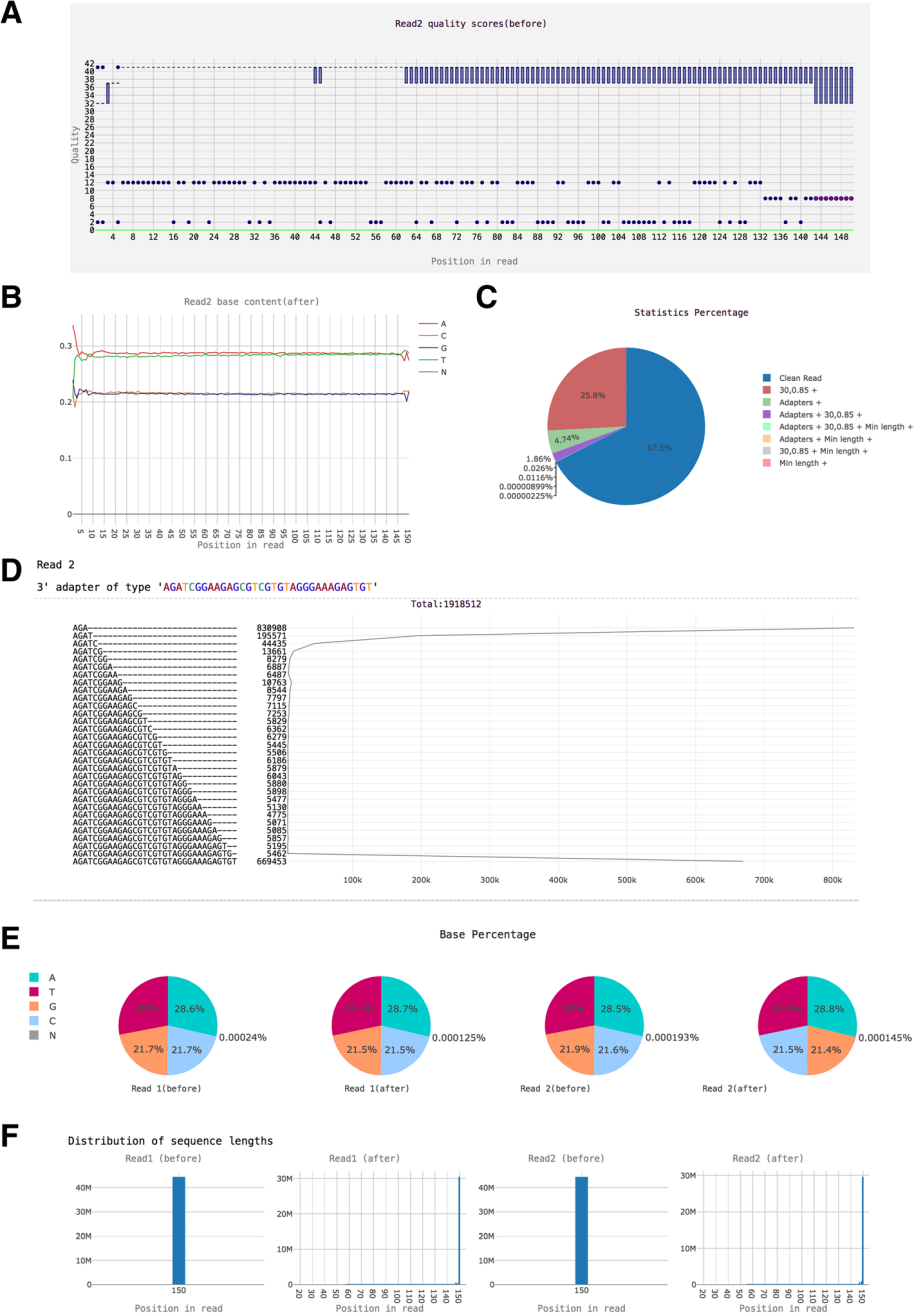
Statistical graphs provided in an HTML report. (**a**). Overview of the range of quality values across all bases at each position in a FASTQ file. (**b**). Proportion of each base at each position in the FASTQ files before and after quality control. (**c**). Percentages of reads filtered by different criterion. ‘+’ indicates reads that were filtered by more than one criterion. (**d**). Number of reads containing adapter sequence at different overlapping bases. (**e**). Percentages of each base in the FASTQ files. (**f**). Length distribution of sequences

We used a paired-end 150-bp whole-genome resequencing data set of soybean to demonstrate the functions of FastProNGS. The total base size of sequencing data was 12.4 GB. The FastProNGS parameters used for the test are shown in the Additional file 1. Parameter “–Q 20” means the quality of bases lower than 20 were trimmed. Parameter “-n” means N bases at the 5′- and 3′-ends were trimmed. If at least three overlapping bases (parameter –O 3) between a read and adapter sequence were found, the overlapping bases will be removed from the read. Parameter “-O” can be set higher than 3 according to the requirements of users. The higher the parameter, the less reads with random adapter matches will be discarded, which may cause lower mapping rate in the downstream analysis. Parameter “-q 30,0.85” means if the percent of bases with more than 30 quality score is less than 85% for one read, the read will be removed. This parameter can be set to a higher value if only highly quality reads are required, such as “-q 30,0.9”. Parameter “-r 0.01” means reads with base N exceeding 1% will be removed. After filtering and trimming, reads with less than 20 bases were removed to improve the efficiency of downstream analyses by parameter “-m 20”. It is recommended to be a lower value in small RNA sequencing projects. Figure 1 shows the summary information provided in the HTML report, such as statistical graphs before and after the filtration.

### Program run-time efficiency

**Fig. 2:**
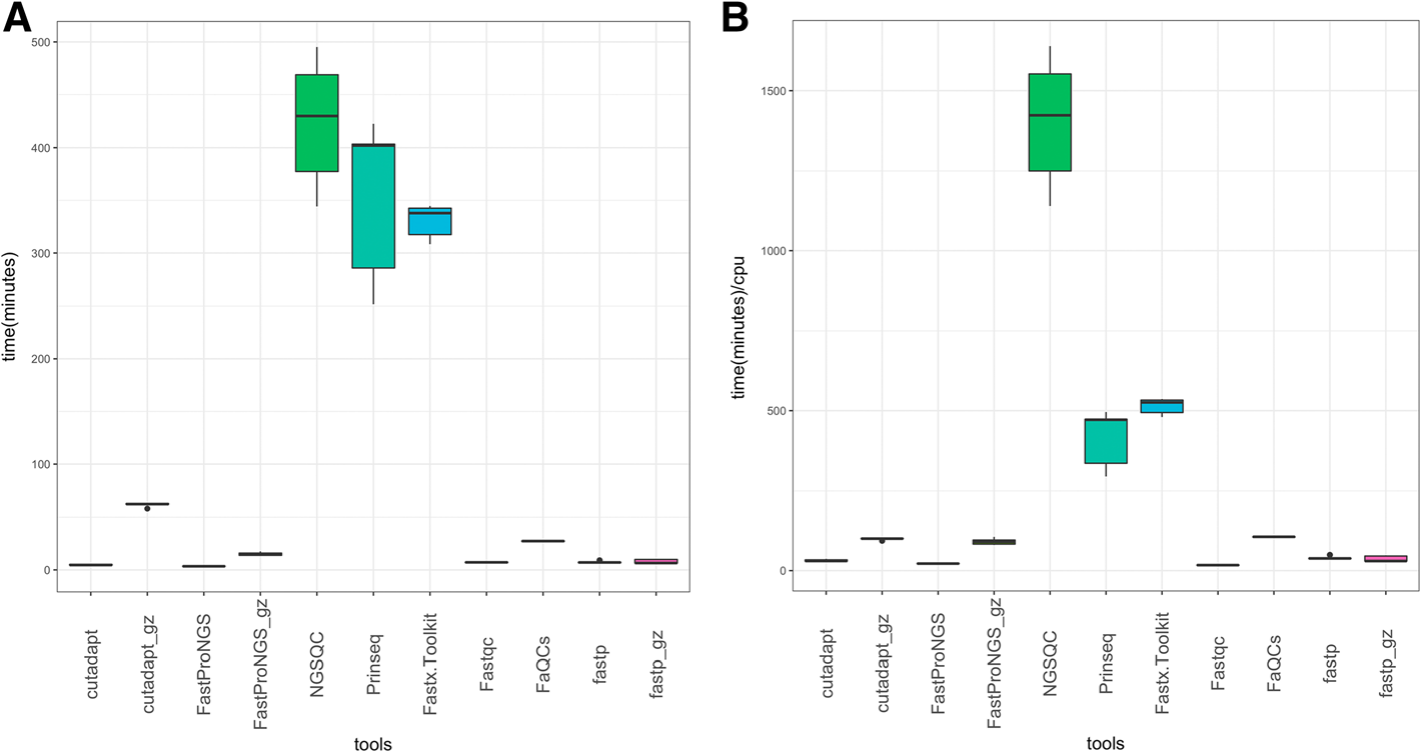
Time costs of different preprocessing tools. The difference between Cutadapt_gz and Cutadapt is whether output files are compressed. Cutadapt_gz indicates the output files were gzip-compressed. **a**. Time cost of different tools using multiple threads. **b**. Time cost of different tools when only one CPU was used

**Fig. 3:**
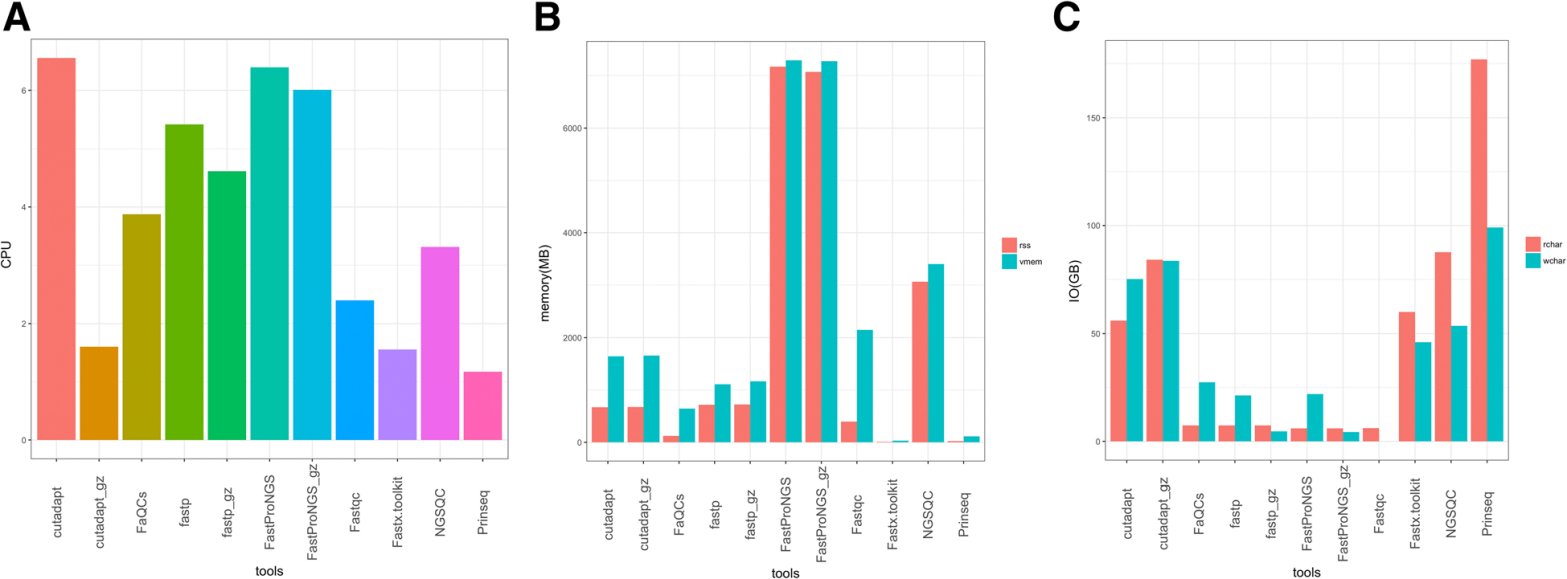
Resources used by different preprocessing tools. **a**. Number of CPUs. **b**. Mean virtual memory size (vmem) and real memory (resident set) size (rss). **c**. Mean number of read and write bytes

To compare the time cost of FastProNGS with existing tools, we first selected tools that performed both QC and adapter removal, namely NGS QC Toolkit (v2.3.3), FASTX-Toolkit (v0.0.14) FaQCs(v2.08) and fastp (v0.19.4). FASTX-Toolkit can only deal with one sequence file at a time, so the total running time is the sum of the processing time of each file. However, commonly a combination of two tools is used to preprocess sequence data; one for QC and the other for adapter removal. Therefore, we also chose PRINSEQ (version 0.20.4) and FastQC (version 0.11.5) as the QC software, Cutadapt (version 1.11) as the adapter removal software. The same dataset of soybean mentioned before was used. For all tools, the closest options for each parameter were used and are listed in Additional file 1. For multi-threaded tools such as FastQC, FaQCs, Fastp, cutadapt and FastProNGS, 8 CPUs were specified. Memory is not restricted. The tasks were run on a computer with 284GB memory and they can use as much memory as desired. All comparisons were run 10 times repeatedly to avoid any instability of the computer system. The real running time, CPU, memory, and IO cost were recorded and the results are shown in Table 2, and Figs. 2 and 3. Because some tools such as PRINSEQ do not support multithreading, we also compared the run-time efficiency where only one CPU was used by multiplying the real running time by the number of CPUs used. In both cases, FastProNGS was faster than the other QC tools except FastQC in the mode of one CPU and fastp if the output files were required to be gzippd. FastProNGS used more memory but much less IO than most tools. For most computers, we believe that the memory used by FastProNGS is small compared with the limited number of CPUs. FastProNGS has the same results with NGS QC Toolkit and Cutadapt respectively (Additional file 2, Figure S1). It should be noticed that FastProNGS and Cutadapt reported about 4% reads containing adapters, but only 1.57 and 0.92% reads were reported to contain adapter sequences by FaQCs and fastp respectively, mainly because they can only trim adapters and don’t allow mismatches and indels in the alignments. The detailed information of the comparisons are shown in Additional file 1.

**Table 2:** Run-time efficiency of tools for processing next-generation sequencing data

Tools	run-time^c^	rss (MB)^d^	vmem (MB)^e^	Average CPU utilization ^f^	rchar(GB)^g^	wchar(GB)^h^	run-time/CPU^i^	Process .gz^j^
NGS QC Toolkit	344	3062	3400	3.312	88	54	1139	R + W
FASTX-Toolkit	308	10	29	1.556	60	46	479	W
PRINSEQ	252	20	115	1.173	177	99	296	–
Fastqc	7	395	2150	2.403	6	0	17	R
Cutadapt^a^	4	670	1645	6.548	56	75	26	R
Cutadapt^b^	58	675	1659	1.605	84	84	93	R + W
FastProNGS^b^	3	7168	7291	6.396	6	22	19	R + W
FastProNGS^a^	13	7066	7270	6.008	6	4	78	R
FaQC	27	121	644	3.876	7	27	105	R
fastp^a^	8	723	1167	4.614	7	5	36	R + W
fastp^b^	7	720	1110	5.421	7	21	40	R

All tools were installed locally and run against the test data set

^a^Output files are gzip-compressed

^b^Output files are not compressed

^c^Minimum task execution time (minutes)

^d^Mean real memory (resident set) size of the process (MB)

^e^Mean virtual memory size of the process (MB)

^f^Average number of CPUs utilized by the process

^g^Number of bytes the process read (GB)

^h^Number of bytes the process wrote (GB)

^i^Minimum task execution time using one CPU (minutes)

^j^Process .gz indicates if the test was natively read (R) or write (W) compressed files. ‘--’ indicates neither read or write compressed files

## Conclusions

With the increasing amount of sequencing data generated per sample, the run-time efficiency is becoming high demanding in data processing. FastProNGS is an easy-to-use stand-alone tool written in C that provides convenient utilities for preprocessing Illumina sequencing data. It supports parallelization to speed up the QC and adapter removal processes by splitting raw data files internally, which saves both time and trivial troubles for users. All the preprocessing functions required for downstream analyses of NGS data can be accomplished using only one command-line. The processing results can be output as plain-text, JSON, or HTML format files, which is suitable for various analysis situations. Ovarall, FastProNGS contains the most comprehensive functions and has advantages in run-time efficiency compared with almost all the existing tools. In the future, we will further optimize FastProNGS, such as improvement in memory usage and run-time efficiency when results are required to be gzipped.

## Availability and requirements

Project name: FastProNGS.

Project home page: https://github.com/Megagenomics/FastProNGS

Operation system: Linux.

Other requirements: libxml2–2.9.7.

Programming languages: C, JAVA.

License: GNU GPL.

Any restrictions to use by non-academics: license needed.

## Additional files

Additional file 1: FastProNGS: Fast Preprocessing for next-generation sequencing reads– Supplementary data FastProNGS Usage (DOCX 107 kb)

Additional file 2: The comparison results of FastProNGS, NGS QC Toolkit and Cutadapt. (PNG 424 kb)

## Data Availability

The datasets supporting the conclusions of this article are included within the article and its Additional file 1. The source codes and small test data of FastProNGS are available at https://github.com/Megagenomics/FastProNGS.

## Abbreviations

NGS: Next Generation Sequence
QC: Quality Control
