# Supplementary material for: FastProNGS: fast preprocessing of next-generation sequencing reads
Source: BMC Bioinformatics. 2019 Jun 17;20:345. doi: 10.1186/s12859-019-2936-9 (PMC6580563; doi:10.1186/s12859-019-2936-9)
Supplement: Supplementary file 1 — FastProNGS: Fast Preprocessing for next-generation sequencing reads– Supplementary data FastProNGS Usage (DOCX 107 kb) [file 12859_2019_2936_MOESM1_ESM.docx]

**FastProNGS: Fast Preprocessing for next-generation sequencing reads– Supplementary data**

**FastProNGS Usage**

Usage: FastProNGS [-agbAGBieOqQlnrmMcEopsxHTjRtwh?v] [file ...]

**-a --adapter** The 3' end adapter (if paired data, represent first read). Supporting anchoring '$'.

**-g --front**  The 5' end adapter (if paired data, represent first read). Supporting anchoring '^'.

**-b --anywhere** The 3' or 5' end adapter (if paired data, represent first read).

**-A --adapter2** The 3' end adapter (if paired data, represent second read). Supporting anchoring '$'.

**-G --front2** The 5' end adapter (if paired data, represent second read). Supporting anchoring '^'.

**-B --anywhere2**  The 3' or 5' end adapter (if paired data, represent second read).

**-i --no-indels** Allow only mismatches in alignments, (Default: allow both mismatches and indels).

**-e --error-rate** Maximum allowed error rate (no. of errors divided by the length of the matching region. Default: see xml).

**-O --overlap** Require MINLENGTH overlap between read and adapter for an adapter to be found, (Default: see xml).

**-q --quality-filter** Discard reads when it less than specific threshold or ratio. (Format: threshold, ratio).

**-Q --quality-low** Cut base less than specific value in anywhere end.

**-n --trim-n** Trim N's on ends of reads (Default: see xml).Don’t use this parameter if you don’t want to trim N.

**-r --ratio-n** Discard reads greater than ratio-n (Default: see xml).

**-m --min-length** Discard reads shorter than LENGTH (Default: see xml).

**-M --max-length** Discard reads longer than LENGTH (Default: see xml).

**-c --gc-content** Discard reads less than specific value (Default: see xml).

**-E --quality-base** quality base (Default: see xml).

**-o --output1** Write trimmed reads1 to FILE.

**-p --output2** Write trimmed reads2 to FILE.

**-s --split** split file count.

**-x –xml**  Default configuration file path

**-H --html** html report file.

**-T --text** text report file.

**-j --json** json report file.

**-R --title** title of report

**-t --threads**  threads of program.

**-k --reads** the num of request read for each process

**-h --help** print help message.

**-v --version** print version message.

**Parameters used in QC softwares**

* $infile1 and $infile2 are two files of the tested paird-end NGS data

**FastProNGS used for test:**

FastProNGS -a AGATCGGAAGAGCACACGTCTGAACTCCAGTCAC -A AGATCGGAAGAGCGTCGTGTAGGGAAAGAGTGT –Q 20 –n –r 0.01 -q 30,0.85 -E 33 –m 20 -o clean.R1.fq -p clean.R2.fq -s 8 -H Result/report.html -t 8 $infile1 $infile2

**Comparing the consistency of QC results between NGS QC Toolkit and** **FastProNGS:**

FastProNGS -q 30,0.85 -E 33 -o clean.R1.fq.gz -p clean.R2.fq.gz -s 8 -H report.html -t 8 $infile1 $infile2 –m 0

**NGS QC Toolkit:**

perl IlluQC_PRLL.pl -pe $infile1 $infile2 N A -l 85 -s 30 -c 8 -o ./ -z g

**Comparing the consistency of cutting adapters results between Cutadapt and** **FastProNGS:**

**FastProNGS:**

FastProNGS -a AGATCGGAAGAGCACACGTCTGAACTCCAGTCAC -A AGATCGGAAGAGCGTCGTGTAGGGAAAGAGTGT -E 33 -o clean.R1.fq -p clean.R2.fq -s 8 -H Result/report.html -t 8 $infile1 $infile2 –m 0 –r 1

**Cutadapt:**

cutadapt -a AGATCGGAAGAGCACACGTCTGAACTCCAGTCAC -A AGATCGGAAGAGCGTCGTGTAGGGAAAGAGTGT -o clean_r1.fq -p clean_r2.fq $infile1 $infile2 -j 8

**Tools used to compare computing resources:**

**FastProNGS:**

FastProNGS -a AGATCGGAAGAGCACACGTCTGAACTCCAGTCAC -A AGATCGGAAGAGCGTCGTGTAGGGAAAGAGTGT -q 30,0.85 -E 33 -o clean.R1.fq -p clean.R2.fq -s 8 -H Result/report.html -t 8 $infile1 $infile2

**FastProNGS_gz:**

FastProNGS -a AGATCGGAAGAGCACACGTCTGAACTCCAGTCAC -A AGATCGGAAGAGCGTCGTGTAGGGAAAGAGTGT -q 30,0.85 -E 33 -o clean.R1.fq.gz -p clean.R2.fq.gz -s 8 -H Result/report.html -t 8 $infile1 $infile2 –m 0

**NGS QC Toolkit:**

perl IlluQC_PRLL.pl -pe $infile1 $infile2 adaptor.txt A -l 85 -s 30 -c 8 -o Result -z g

**cutadapt:**

cutadapt -a AGATCGGAAGAGCACACGTCTGAACTCCAGTCAC -A AGATCGGAAGAGCGTCGTGTAGGGAAAGAGTGT -o Result/clean_r1.fq -p Result/clean_r2.fq $infile1 $infile2 -j 8

**cutadapt_gz:**

cutadapt -a AGATCGGAAGAGCACACGTCTGAACTCCAGTCAC -A AGATCGGAAGAGCGTCGTGTAGGGAAAGAGTGT -o Result/clean_r1.fq.gz -p Result/clean_r2.fq.gz $infile1 $infile2 -j 8

**Fastx_toolkit:**

fastx_clipper -a AGATCGGAAGAGCGTCGTGTAGGGAAAGAGTGT -l 80 -n -z -i $outfile1 -o $outfile2

**PRINSEQ:**

perl prinseq-lite.pl -verbose -fastq $infile1 -fastq2 $infile2 -out_good file_passed -out_bad file_filtered -log log.txt -min_qual_mean 30 -min_len 80

**FaQCs**

FaQCs -1 $infile1 -2 $infile2 --adapter --artifactFile adapter.txt --ascii 33 -t 8 --avg_q 30 -d ./

**Fastqc:**

fastqc -o . -t 8 –a adapter.txt $infile1 $infile2

**fastq:**

/Mega/bioinfo/PMO/liuxiaoshuang/project/FPNGS/software/fastp -i $infile1 -o clean.R1.fq.gz -I $infile2 -O clean.R2.fq.gz --adapter_sequence AGATCGGAAGAGCACACGTCTGAACTCCAGTCAC --adapter_sequence_r2 AGATCGGAAGAGCGTCGTGTAGGGAAAGAGTGT --unqualified_percent_limit 15 -q 30 -s 8 -w 8

**The consistency results of NGS QC Toolkit, FastProNGS and Cutadapt**

Figure S1. The statistics results of FastProNGS (A1,A2) and NGS QC toolkit (B1,B2). A1 and B1 showed the same number of reads and bases before and after the filtration. A2 and B2 showed the same percentages of base A,T,C,G and N before and after QC.

Table S1. The statistics results of FastProNGS and Cutadapt.

|  | FastProNGS | Cutadapt |
| --- | --- | --- |
| Total read pairs processed | 44471264 | 44471264 |
| Read 1 with adapter | 1656176 (3.72%) | 1,656,176 (3.7%) |
| Read 2 with adapter | 1918512 (4.31%) | 1,918,512 (4.3%) |
