# Supplementary figures and images for: FastProNGS: fast preprocessing of next-generation sequencing reads
Source: BMC Bioinformatics. 2019 Jun 17;20:345. doi: 10.1186/s12859-019-2936-9 (PMC6580563; doi:10.1186/s12859-019-2936-9)

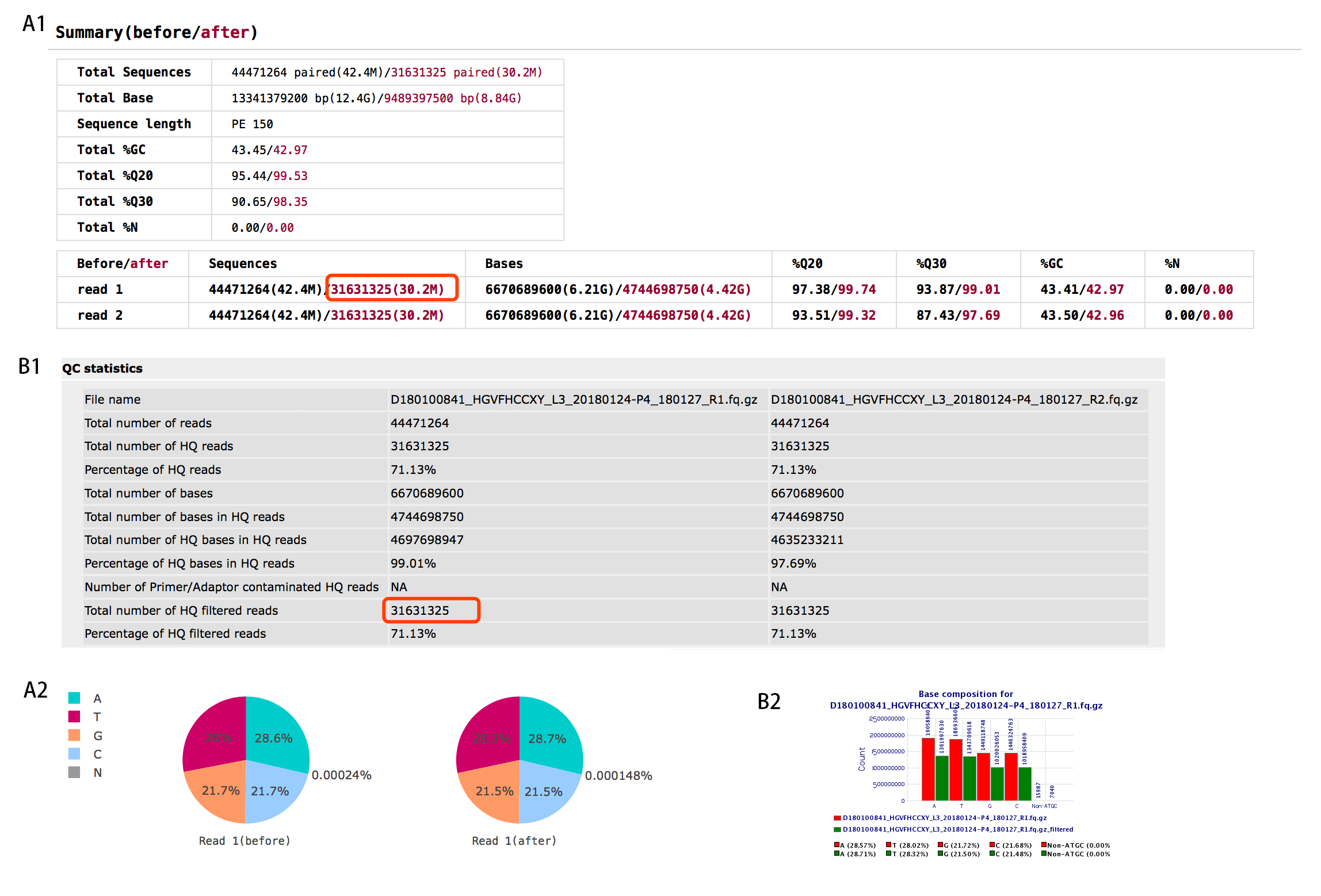

Supplement: Supplementary file 2 — The comparison results of FastProNGS, NGS QC Toolkit and Cutadapt. (PNG 424 kb) [file 12859_2019_2936_MOESM2_ESM.png]
